# Hepatitis C virus as a systemic disease: reaching beyond the liver

**DOI:** 10.1007/s12072-015-9684-3

**Published:** 2015-12-10

**Authors:** Kirat Gill, Hasmik Ghazinian, Richard Manch, Robert Gish

**Affiliations:** Department of Internal Medicine, St. Joseph’s Hospital and Medical Center, Phoenix, AZ USA; Hepatology Department, Nork-Marash Medical Center, 13 Armenak Armenakyan Street, 0047 Yerevan, Armenia; Department of Infectious Disease, Nork-Marash Medical Center, 13 Armenak Armenakyan Street, 0047 Yerevan, Armenia; Department of Medicine, Division of Gastroenterology and Hepatology, Stanford University, Stanford, CA USA; National Viral Hepatitis Roundtable, San Francisco, CA USA

**Keywords:** Extrahepatic manifestations, Chronic hepatitis C, Mixed cryoglobulinemia, Insulin resistance, Diabetes mellitus, Lymphoproliferative disorder, HCV brain syndrome, Renal failure

## Abstract

Chronic hepatitis C (CHC) is associated with multiple extrahepatic manifestations that may impact infected patients. The mechanisms through which these develop include those which are immunological, in which the chronic persistence of virus leads to the circulation of immune complexes (mixed cryoglobulinemia) and other autoimmune phenomena, and those which are virological and related to the extrahepatic tropism of the virus to other tissues. It is estimated that 40–74 % of patients with CHC may develop at least one extrahepatic manifestation during the course of the disease. Extrahepatic syndromes may represent the first signal of hepatitis C infection in some patients. CHC is associated with a four-fold increased risk of insulin resistance and type 2 diabetes mellitus; with cardiovascular disease in 17–37 % of patients; and with increased risk for cerebrovascular deaths, with a biological gradient of cerebrovascular mortality correlating with an increasing serum viral load. CHC is also associated with lymphoproliferative disorders, particularly non-Hodgkin B-cell lymphoma. The kidney is involved in 35–60 % of patients with CHC-associated mixed cryoglobulinemia. The prevalent type of glomerulonephritis associated with mixed cryoglobulinemia is membranoproliferative glomerulonephritis. In 30 % of cases, renal involvement begins with a nephritis syndrome and acute renal failure, while in 55 % there is only mild hematuria, microalbuminuria, proteinuria and renal insufficiency. CHC is also associated with cognitive impairment, especially in memory and concentration. Thus, extrahepatic CHC manifestations involve multiple organ systems outside the liver linked to a variety of comorbidities which may lead to significantly increased mortality from non-liver-related events.

## Introduction

The hepatitis C virus (HCV) has significant and obvious hepatic implications. However, too often, the extrahepatic, multi-organ ramifications of this virus are overlooked. Due to the fact that HCV has been shown to infect both hepatocytes and lymphocytes, lymphoproliferative diseases such as lymphoma and mixed cryoglobulinemia (MC) are most closely linked to hepatitis C infection [1]. However, HCV has also been implicated in derangements of multiple other organ systems including the integumentary, ocular, muscular, skeletal, nervous, endocrine, cardiovascular, respiratory, and urinary systems. Furthermore, chronic hepatitis C (CHC) has a crippling and debilitating psychosocial effect. Clinical vigilance and acumen are needed to recognize these extrahepatic manifestations (EMs) as these may often be the initial findings in a patient previously undiagnosed with HCV infection. Studies have demonstrated that up to 74 % of patients show at least one EM [2].

The EMs can be divided into those with a high degree of association with HCV and those with a more moderate or mild association [3]. Some of the extrahepatic manifestations, including MC and non-Hodgkin B-cell lymphoma, have significant prevalence and clear data supporting a causal relationship. Other manifestations have been noted to have a higher prevalence compared to controls in trials. These include dermatological findings (porphyria cutanea tarda), fatigue, cardiovascular disease (stroke, coronary artery disease), renal involvement, and neurological disorders (depression, impaired quality of life) [4].

Certain extrahepatic manifestations are known to occur in patients with MC. These include palpable purpura, vasculitis, arthralgias, and nephropathy [3]. However, these manifestations have also been reported without MC. For example, patients with CHC have developed membranous nephropathy in the absence of cryoglobulins or evidence of MC. Patients with MC will often have elevated Rheumatoid Factors and low complement levels which may serve as a means to differentiate manifestations occurring secondary to MC [3].

## Cryoglobulinemia and immune complex disease

Cryoglobulins are immunoglobulins that precipitate in vitro at temperatures less than 37 °C and often produce organ damage via either a hyperviscosity syndrome or immune-mediated mechanism [5]. Although cryoglobulinemia can be caused by a wide variety of infections and disorders, CHC is known to be the most common cause [6]. Three basic types of cryoglobulinemias are recognized according to the clonality and type of immunoglobulins. Type I consists of monoclonal immunoglobulins, generally either IgM or IgG. Types II and III are often referred to as mixed cryoglobulinemia (MC). Type II cryoglobulins are a mixture of monoclonal IgM and polyclonal IgG. Type III is a mixture of polyclonal IgM and IgG. CHC is primarily associated with type II cryoglobulinemia [5] in which the primary mechanism of injury is vasculitis that occurs via immune complex deposition. The immunoglobulins produce immune complexes, which in turn bind to the receptors on endothelial cells, allowing for subsequent deposition and inflammation [5]. The vessels involved are primarily small vessels and histopathologically it is known as leukocytoclastic vasculitis.

Although the pathogenesis is not entirely understood, cryoglobulinemia is markedly common in those with CHC. In two separate studies, Lunel et al. and Pawlotsky et al. found that 36–54 % of patients with CHC had detectable cryoglobulins [7, 8]. Lunel et al. showed that 25 % of patients with CHC and cryoglobulins displayed clinical symptoms which could be related to cryoglobulinemia [7]. Few organs are left unaffected by immune complex deposition, with vasculitis occurring in the skin, joints, kidneys, lungs, heart, digestive tract, and brain. In an Iranian population, Jadali et al. found autoimmune disorders associated with CHC that include Sjögren syndrome, thyroid disease, systemic lupus erythematosus, immune thrombocytopenic purpura, and rheumatoid arthritis [9]. Patients with MC are also predisposed to malignancies, specifically lymphoma as well as IgM paraproteinemia and low-grade lymphoma, a syndrome known as Waldenstrom macroglobulinemia [10]. Involvement with one or multiple organs can present as a life-threatening manifestation. Because MC occurs as a sequela of CHC, treatment is aimed at the underlying disease process.

## Renal involvement

Renal involvement is the most common severe manifestation of MC [11]. Given the increased propensity to develop MC and subsequently renal disease, patients with CHC are more likely to develop end-stage renal disease than the general population [12]. Although other renal diseases have been associated with CHC, chronic kidney disease occurs most often in the context of MC [13]. However, direct protein deposition of HCV RNA and associated proteins has also been implicated in renal disease [13]. Renal manifestations of CHC include Type I membranoproliferative glomerulonephritis (MPGN), mesangial glomerulonephritis and focal and segmental glomerulonephritis. In a China-based study, minimal change nephropathy and membranous nephropathy were reported by Cheng et al. [1]. Fibrillary glomerulonephritis, immunotactoid glomerulopathy, IgA nephropathy, renal thrombotic microangiopathy, vasculitic renal involvement and interstitial nephritis have been reported in a Malaysian population [12]. In one study of patients with CHC who underwent biopsy, type I MPGN was the most common finding associated with MC [11]. Ultimately, treatment is directed at the underlying virus. Given the potentially fatal sequelae of the aforementioned diseases, many clinicians recommend annual screening for microalbuminuria, microscopic hematuria, and cryoglobulinemia in patients with CHC [12].

## Dermatological manifestations

Cacoub et al. found that 17 % of those with CHC displayed a dermatological disorder, the most common being purpura (7 %) and Raynaud’s phenomenon (7 %) [2]. Palpable purpura, a non-blanching skin disorder, is the most common clinical manifestation of MC, occurring in up to 90 % of cases [14]. Purpura, caused by an extravasation of red blood cells into the dermis, is usually relegated to the buttocks and lower extremities, with the face usually spared. When small-to-medium-sized arterial vessels are involved, polyarteritis nodosa may occur leading to an infarct of the skin and subsequent irregular, often painful, skin lesions [15]. Other immune complex-mediated skin disorders include Raynaud’s phenomenon, which is characterized by intermittent vasospasm triggered primarily by cold environment or emotional stress. The vasospasm causes cyanosis, blanching and often pain of the distal digits. It can occur in up to one-third of cases and primarily involves the hands, feet, lips, ears, and the tip of the nose [14].

Many other skin disorders have been associated with CHC, some with better established causality than others. These disorders include porphyria cutanea tarda, lichen planus, necrolytic acral erythema, erythema multiforme, and pruritus. Less commonly reported skin manifestations include psoriasis and sarcoidosis. Porphyria cutanea tarda, caused by deficiency of hepatic uroporphyrinogen decarboxylase, is the most common porphyria. It is characterized by painful vesicular lesions on sun-exposed regions of the skin which rupture and eventually scar [15]. Lichen planus, a mucocutaneous disorder, is also strongly associated with CHC. Gandolfo et al. showed that, of their patients with oral lichen planus, 62 % had HCV antibodies and 60 % had HCV RNA [16]. A meta-analysis of 36 studies regarding lichen planus and HCV showed that there was also an increased risk of having lichen planus among CHC patients [17]. Chronic lichen planus is considered a premalignant condition, especially when involving the oral cavity, and has the propensity to progress to squamous cell carcinoma. Although data are limited on the effect of the new direct acting antivirals on the cutaneous manifestations of CHC, there is evidence from earlier studies that dermatological EMs including porphyria cutanea tarda often resolve with treatment with pegylated interferon (peg-IFN) and ribavirin [18–20]. Data on the treatment effect on lichen planus, however, are conflicting, with both worsening of manifestations and regression having been reported [20].

Necrolytic acral erythema is thought to be the dermatological disorder that is most strongly associated with CHC and is often considered to be pathognomonic for CHC. It is characterized by painful or pruritic, erythematous skin lesions involving acral skin surfaces [21]. Although the etiology is not entirely clear, necrolytic acral erythema has been shown to improve with administration of zinc [22]. Therefore, impairment in the regulation of zinc, which can occur in CHC, is thought to be linked to the pathogenesis [23].

## Ocular manifestations

Several ocular manifestations that are secondary to immunological mechanisms have been associated with HCV. Disorders involving the anterior segment of the eye include dry eye, Mooren ulcer, scleritis and episcleritis, trichomegaly, and peripheral ulcerative keratopathy among others. Posterior segment involvement can lead to multiple disorders including cotton wool spots, retinopathy, central vein thrombosis, crystal macular edema, nonarteritic anterior ischemic optic neuropathy, and optic neuropathy [24]. Of these, dry eye syndrome and ischemic retinopathy seem to be the most common. Mooren ulcer is a rare ocular complication associated with CHC. It is characterized by progressive stromal ulceration. Although the pathogenesis is not completely understood, it is thought to be an autoimmune process against target antigens in the corneal stroma [24].

## Metabolic manifestations: diabetes and insulin resistance

Metabolic disorders have been associated with CHC. Data from the 1988 to 1994 NHANES cycle showed an independent association of insulin resistance and diabetes with HCV infection. In later NHANES cycles (1998–2008), these associations were no longer found to be significant; however, the relationship may have been diluted by the increase of other risks for diabetes, most importantly the rapid increase in the prevalence of obesity [25]. Multiple other studies have shown a causal relationship between CHC and insulin resistance, type 2 diabetes and subsequent steatosis [26, 27]. Cheng et al. found that type 2 diabetes mellitus was seen in 28 % of CHC patients, a significantly higher prevalence compared to the study’s general population [1]. Mehta et al. showed that CHC patients who were at high risk for diabetes were more than 11 times as likely as those without CHC to develop diabetes [28]. Another study found that CHC is an independent predictor of diabetes [27]. Even with adjustment for other major predisposing conditions such as older age, obesity, and smoking, CHC was associated with insulin resistance and diabetes. In a large longitudinal study that followed 4958 persons from a community-wide cohort in southern Taiwan for 7 years, it was found that CHC, coinfection with hepatitis B virus, overweight, obesity, and increasing age were significantly associated with diabetes development [29]. Because with stratification by age and body mass index the risk ratio for diabetes in CHC participants increased when age decreased and body mass index levels increased, the researchers concluded that HCV infection is an independent predictor of diabetes, especially for CHC persons who are younger or have a higher body mass index. In patients treated with peg-IFN and ribavirin, it was found that in patients with pre-treatment insulin resistance, insulin sensitivity was improved at 12 weeks and 24 weeks, and at end of therapy (24 or 48 weeks) [30]. The findings of both cross-sectional and longitudinal studies [31] and a large meta-analysis [32] have suggested a direct role of HCV in inducing derangement of glucose metabolism. The mechanism of action is thought to be secondary to an effect on glucose-insulin regulation, and lipid metabolism and synthesis [27].

## Salivary gland

Sjögren syndrome is described as a chronic, slowly progressive autoimmune disease characterized by lymphocytic infiltration of the exocrine glands and B-lymphocyte reactivity resulting in xerostomia and dry eyes. Histological changes characteristic of Sjögren syndrome, focal lymphocytic sialadenitis, were significantly more common in CHC patients [33]. It is not entirely clear whether HCV causes a Sjögren-like illness or is responsible for the onset of Sjögren. Although sialadenitis is common, dry eyes and dry mouth are often absent. Furthermore, although Sjögren syndrome causes periductal lymphocytic infiltration, CHC patients are noted to have pericapillary lymphocytic infiltration [34]. Lymphoma is a well-known manifestation of Sjögren syndrome that usually presents later in the illness. Most lymphomas are extranodal, low-grade marginal zone B-cell lymphomas [15].

## Malignancies

Several viruses have been linked to the development of cancer. It has been estimated that 18.6 % of cancers worldwide are attributable to five infections: *Helicobacter pylori* (37.0 %), human papillomavirus (27.9 %), HCV plus hepatitis B virus (24.8 %), and Epstein–Barr virus (10.3 %) [35]. The burden of cancers related to organisms is clearly more apparent in developing countries as compared to developed nations. If infections linked to cancer were eradicated there would be 26.3 % fewer cancers in developing countries (1.5 million cases per year) and 7.7 % fewer in developed countries (390,000 cases) [36].

Although hepatocellular carcinoma is the cancer most commonly associated with HCV, it has been found that several other malignancies are more common in those with CHC compared to the general population. Prostate cancer has been noted to correlate with detection of hepatitis C antibody [37]. In a study of Japanese patients, both oral and other digestive tract cancers were shown to correlate with HCV infection [38]. As previously stated, squamous cell carcinoma is associated with lichen planus. However, oral verrucous and squamous cell carcinoma were reported in CHC patients even without the presence of oral lichen planus [34]. In patients with MC, CHC and autoimmune thyroiditis, there is an increased prevalence of papillary thyroid cancer [39].

The strongest association of CHC and cancers other than HCC is with lymphoma, specifically diffuse large B-cell non-Hodgkin lymphoma, the most common subtype of lymphoma in the USA [40]. Due to the fact that tumor regression occurs when antiviral therapy begins, it is thought that CHC is directly responsible for tumor progression although the mechanism of how this occurs is not entirely understood. Studies have also assessed the possibility of an association between HCV and breast cancer. In a large, population-based study in Taiwan, no significant association was found between breast cancer risk and HCV seropositivity; however, an age-stratification analysis conducted by Su et al. in Taiwan found that HCV patients aged less than 50 years had a two-fold greater risk of breast cancer [41]. In a smaller study in France, chronic HCV infection was not found to be a promoter of breast cancer in adult females of any age [42].

## Pulmonary fibrosis

Pulmonary fibrosis, manifesting most commonly as dyspnea on exertion and nonproductive cough, is histologically characterized by interstitial inflammation, foci of proliferating fibroblasts, and dense collagen fibrosis [15]. Although the pathogenesis is not clear, several studies have shown a link between CHC and the development of pulmonary fibrosis. A study of Japanese patients compared those with CHC, chronic hepatitis B and a control group. It was found that there was a higher prevalence of pulmonary fibrosis in those with CHC compared to the other two groups [43]. In another study, patients with a diagnosis of idiopathic pulmonary fibrosis (IPF) were tested for antibodies to hepatitis C virus via an enzyme-linked immunosorbent assay (ELISA). More than 25 % were positive for the test and the rate of CHC was higher in the patients with idiopathic pulmonary fibrosis compared to those who did not have CHC [44]. Another study showed similar results with those patients testing positive for HCV having an increasing incidence of lung disease, the most common being IPF [44].

## Psychiatric manifestations

Patients with CHC have a higher prevalence of both mental illness and substance abuse compared with the general population [45, 46]. Given this, there have been recommendations to screen patients with substance abuse disorders and/or mental illness such as bipolar disorder, as the relative risk for CHC is significantly higher than in those without these conditions [47]. Although improving over time, there is still a stigma associated with a diagnosis of hepatitis C. In turn, patients may have feelings of embarrassment or shame leading to the delay or avoidance of treatment. Feelings of depression or anxiety often accompany the diagnosis. Given the increased prevalence of drug abuse in the mentally ill population [48], it is hard to assess whether depression is a sequela of the disease or a predisposing factor. Furthermore, treatment compliance and disease management are often compromised given the underlying mental illness.

Studies have shown that patients with CHC are more likely to have depression and an impaired quality of life [49, 50]. In those who do seek treatment, achieving a 12-week sustained viral response (SVR) was found to be protective for depression [51]. Furthermore, it was found that patients with SVR had a better quality of life than those who failed treatment [52]. Psychosocial assessment is a crucial step in the overall management of CHC and is too often overlooked. This assessment is clearly important for quality of life and treatment compliance as psychosocial impairment in HCV patients significantly affects treatment adherence [53].

## Thyroid involvement

Mao et al. reported that, in Chinese patients, thyroid dysfunction, including chronic thyroiditis, hypothyroidism and hyperthyroidism, was associated with CHC [54]. The etiology is primarily autoimmune and the prevalence of anti-thyroid autoantibodies ranges from 4 to 15 % in CHC infection [55]. Thyroid abnormalities, primarily Hashimoto’s disease and isolated increases of anti-thyroid antibodies, appear to be more frequent in chronic hepatitis C than in chronic hepatitis B or D [56]. Patients with MC and CHC were more likely to have increased levels of serum anti-thyroperoxidase autoantibody and anti-thyroglobulin autoantibody than the general population. This is thought to occur via a complex mechanism involving T helper cells [39]. Patients with CHC are more susceptible than patients with chronic hepatitis B to autoimmune thyroid disease. Screening of thyroid gland function and an antibody panel are recommended by some clinicians for all patients with CHC [57].

## Cardiovascular disease

According to the World Health Organization, ischemic heart disease and stroke are the two leading causes of death worldwide [58]. Coronary artery disease (CAD), peripheral arterial disease (PAD) and cerebrovascular disease have all been shown to be increased in those with CHC. Although a recent systematic review has shown that the association remains inconclusive, warranting the need for further research [59], multiple studies including those in Asian populations have shown that there is an increased risk of stroke in those with CHC [60, 61] and CHC treatment appears to decrease this risk [62]. In a study based in Taiwan, there was an increased risk of stroke with increasing serum HCV RNA levels; there was no association between virus genotypes and risk [63]. Localization of HCV RNA in human carotid plaques provides strong evidence for an association between HCV and atherosclerosis and suggests that the HCV RNA in plaque tissue is an active local infection linked to tissue damage [64]. Inflammation and replication of the virus within both carotid plaques and brain endothelial cells have been found and are thought to be key mediators [65]. CHC has been shown to increase carotid thickness and/or plaque as seen on ultrasonography [63].

Although CHC patients are less likely to have hypertension and have lower mean total cholesterol, low-density lipoprotein cholesterol and triglycerides, many studies have shown that patients have an increased risk of CAD despite this favorable risk profile [66, 67]. A Turkish study by Alyan et al. involving patients with known CAD showed that CHC patients had a higher level of C-reactive protein and fibrinogen and that infection with the virus was an independent predictor for severity of CAD [68]. A Taiwanese study showed that patients with CHC had an almost 1.5-fold higher risk of developing PAD than those without HCV infection [69]. This number increased in patients with increasing age and comorbidities such as chronic kidney disease. Patients who were aged 65 and older had an almost 12-fold higher risk of developing PAD. Congestive heart failure as a subtype of cardiovascular disease was also shown to be increased in those with CHC. In another study, patients with concomitant CHC and congestive heart failure were significantly younger than average for the population [27]. In addition, patients with CHC have been shown to have increased pulmonary vascular resistance and pulmonary systolic pressure [70].

## Neuropathies

Peripheral nerves are composed of sensory, motor, and autonomic elements. Thus, peripheral neuropathies can impair sensory, motor or autonomic function either singly or in combination [15]. Peripheral neuropathies are further classified into those that primarily affect the cell body, myelin, and the axon [15]. HCV can affect all three portions of the neuron, with CHC patients experiencing axonal neuropathies such as sensorimotor polyneuropathy, large or small fiber sensory neuropathy, motor polyneuropathy, and mononeuritis, among others. Demyelinating disorders including chronic inflammatory demyelinating polyneuropathy, the Lewis-Summer Syndrome, and cryoglobulin-associated polyneuropathy are also associated with CHC [71].

CHC patients have been shown to have a higher prevalence of sensory, motor and autonomic dysregulation than the general population. The etiology is not entirely clear. Some postulated causes include vitamin deficiency, cryoglobulinemia and immune complex deposition, and metabolic conditions such as diabetes which CHC patients have an increased propensity to develop. Although alcoholism and substance abuse are more common in those with CHC and levels of B12 tend to be lower in these groups of patients, one Egyptian study specifically showed that neuropathy was not related to B12 and CHC was an independent risk factor [72]. Another study showed that peripheral neuropathy associated with mixed cryoglobulinemia secondary to CHC was related to the duration of HCV infection and unrelated to the duration of cryoglobulinemia [73].

## Neurologic disorders

Similar to the other extrahepatic manifestations mentioned, neurological manifestations are thought to be immune mediated but may also occur through invasion of the virus in neural tissue [71]. Researchers who have reported that HCV sequences are detected in cerebrospinal fluid (CSF) have noted that, in patients who have different virus strains in serum and peripheral blood mononuclear cells (PBMC), the CSF-derived virus is similar to that found in PBMC, leading to the hypothesis that PBMC might carry HCV into the brain [74]. Although most research has focused on HCV replication in hepatocytes, several studies have provided evidence that HCV replication occurs in the central nervous system, although probably at considerably lower levels than in the liver [75, 76].

Negative-strand HCV RNA has been found in autopsy brain tissue samples, with serum- and brain-derived viral sequences shown to be different [75]. Furthermore, HCV receptor expression is not exclusive to hepatocytes. It has been reported that brain microvascular endothelial cells, the major component of the blood–brain barrier, express all receptors required for HCV entry [77]. In a study of autopsy brain tissue, the brain cells that were found to harbor HCV were mainly CD68+ cells (macrophages/microglia) and, to a lesser extent, astrocytes [78].

The physical sequelae of CHC are often grossly apparent but the cognitive dysfunction associated with this disease is often missed. CHC has been associated with a poorer quality of life secondary to neuropsychiatric and psychosocial problems, leading often to difficulties in management and subsequently in the course of the disease. A wide variety of neurological manifestations have been shown to occur secondary to CHC. Cognitive impairment, particularly in power of concentration and speed of working memory, that is unaccounted for by depression, fatigue, or a history of injection drug use has been reported in CHC patients [79]. In their Taiwan-based study, Chiu et al. found an increased incidence of dementia in those with CHC after adjustment for variables such as hepatic encephalopathy and alcohol-related disease [80]. Another study showed executive functions (performance on clock drawing study, digit span backward test, animal verbal fluency test, and EXIT 25 test) to be impaired in those with CHC [81]. In one study, a neurocognitive test, the Repeatable Battery for the Assessment of Neuropsychological Status, was administered to inmates with CHC to assess cognitive functioning. Compared with the control group, those with CHC performed at a significantly lower level [82]. A study involving veterans showed that, when comorbidities and substance abuse disorders were accounted for, there were significant differences in memory, attention, processing of information and reasoning in CHC patients [83].

## Treatment effect

Although data are limited on the resolution of extrahepatic manifestations with the new direct acting antiviral medications, multiple studies over the course of the past few decades have shown marked improvement of EMs upon eradication of HCV after treatment with previous therapies, primarily combination therapy with peg-IFN and ribavirin. With standard courses of peg-IFN/ribavirin the following have been seen:

- Reduced insulin resistance, with improved insulin sensitivity seen at 12 weeks and 24 weeks, and at end of therapy (24 or 48 weeks) [30].
- Improved health-related quality of life [52, 84].
- Improved work productivity [84].
- Reduced depression [51].
- Resolution of dermatological EMs including porphyria cutanea tarda [18–20] and lichen planus, although data on the latter are conflicting with both worsening and regression having been reported [20].
- Reduced stroke risk [62].
- Clearance of cryoglobulins with reduced risk of end organ damage [85].

Emerging data on treatment with the direct acting antivirals show similar effects. A recent study showed decreased fatigue at SVR 12 compared to baseline with treatment with ledipasvir and sofosbuvir [86]. Viral eradication with sofosbuvir/ledipasvir has also been associated with improvement in health-related quality of life and work productivity [87].

## Conclusion

Although commonly having the connotation of being a disease of the liver, chronic infection with HCV has the ability to cause significant extrahepatic manifestations affecting virtually every organ via a variety of mechanisms. Awareness on the clinician’s part is necessary to recognize these sequelae. This in turn can lead to appropriate screening, early treatment and improved outcomes. These systemic manifestations of CHC may explain the increased all-cause mortality that is seen even when excluding liver disease-associated mortality.
